# Protective Effect of Leaves of *Murraya koenigii *on Reserpine-Induced Orofacial Dyskinesia

**Published:** 2012

**Authors:** Rupali Patil, Kiran Dhawale, Hanmant Gound, Rajendra Gadakh

**Affiliations:** *Department of Pharmacology, MGV’s Pharmacy College, Panchavati, Nashik-422 003, Maharashtra, India.*

**Keywords:** Vacuous chewing movements, Tongue protrusions, Free radicals, Murraya koenigii

## Abstract

*Murraya koenigii *L. (Rutaceae), commonly known as curry leaf tree, closely associated with south India where the word “curry” originates from the Tamil “kari” for spiced sauces. Curry leaves are a rich source of carbazole alkaloids which possess various biological activities such as antitumor, antioxidant and anti-inflammatory. Curry leaf has a potential role in the treatment of diabetes. Reserpine-induced orofacial dyskinesia in rats is an animal model of tardive dyskinesia that has been linked with free radical generation and oxidative stress. In this study, neuroprotective potential and *in-vivo* antioxidant status of methanol extract of the leaves of *Murraya koenigii* (MEMK) in reserpine-induced orofacial dyskinesia are investigated. Reserpine was used to induce orofacial dyskinesia. The effect of MEMK on locomotion and catalepsy was studied using Open-field apparatus and Bar-test, respectively. The effect of MEMK on the levels of protective anti-oxidant enzymes *i.e*. superoxide dismutase (SOD), catalase (CAT), glutathione reductase (GSH) and inhibited lipid peroxidation (LPO) in forebrain region were investigated in reserpine-treated animals. Results demonstrated that the MEMK significantly inhibited the reserpine-induced vacuous chewing movements (VCM), tongue protrusion (TP), orofacial burst (OB) and catalepsy. MEMK significantly increased the number of squares traversed and rearing in open field apparatus. Treatment with MEMK significantly restored the levels of protective anti-oxidant enzymes *i.e.* SOD, CAT, GSH and inhibited LPO in forebrain region when compared with reserpine. It also inhibited haloperidol-induced catalepsy. The present study concludes that the oxidative stress might play an important role in reserpine-induced abnormal oral movements, and *Murraya koenigii* may have great potential in the treatment of neuroleptic-induced orofacial dyskinesia

## Introduction

The central nervous system is especially vulnerable to free radical damage because of the brain’s high oxygen consumption, its abundant lipid content, and the relative paucity of antioxidant enzymes as compared with other tissues (1). The neurodegenerative hypothesis suggests that persistent tardive dyskinesia (TD) may be associated with, or induced by, neuronal damage somewhere in the basal ganglia. Neuroleptics damage the γ-amino butyric acid (GABA) neurons in the basal ganglia. These damaged neurons in turn release the inhibitory effects that GABA neurons have on the dopaminergic output neurons in the nigrostriatal tract, possibly leading to a state of dopamine excess or hyperfunction in the basal ganglia. Building on this hypothesis, Cadet and Lohr (1989) proposed that the damage, which could affect the neuronal membrane function and, if unchecked, could lead to cell death, might involve the presynaptic catecholaminergic fibers as well as other neurotransmitter systems. These neuronal changes were secondary to the free radical generation from the increased dopamine metabolism and turnover, a consequence of neuroleptic medication (2). The increased dopamine turnover caused by neuroleptics could lead to excessive production of these potentially damaging free radicals. *Murraya koenigii* L. (Rutaceae) is known to have good antioxidant potential (3).

Hence, in the present study we explored the potential of *Murraya koenigii* through the use of behavioral and biochemical models of orofacial dyskinesia in rats. In the present study, we investigated the effect of MEMK on reserpine-induced orofacial dyskinesia. Also the effect of MEMK on locomotor activity and biochemical parameters like protective anti-oxidant enzyme levels (SOD and CAT), GSH levels and LPO in the forebrain region were studied in reserpine treated rats. The effect of MEMK on the haloperidol-induced catalepsy was studied in mice.

## Experimental

*Animals*

Male albino mice (Swiss: 22-25 g) and male albino rats (Wistar strain: 150-200 g) were obtained from Bharat Serum and Vaccine Ltd., Thane. Animals were housed in groups of five at ambient temperature of 25 ± 1°C. Animals had free access to water and food. They were deprived of food but not water 4 h before the experiment. The experiments were carried out between 9.00 and 14.00. All the experimental procedures and protocols used in this study were approved by the Institutional Animal Ethics Committee (IAEC) of M.G.V’s Pharmacy College, Panchavati, Nashik, constituted under the provisions of Committee for Purpose of Control and Supervision of Experiments on Animals (CPCSEA), Ministry of Environment and Forests, Government of India. Ethical guidelines were strictly followed during the experiments.

**Table 1:** Effect of MEMK on reserpine-induced orofacial dyskinesia in rats.

**Treatment (mg/Kg, p.o.)**	**VCM**	**OB**	**TP**
**Vehicle**	12.25 ± 0.62	6 ± 0.70	2.75 ± 0.47
**RE (1)**	166 ± 2.52^#^	41.5 ±2.02^#^	12 ± 0.70^#^
**MEMK (200)**	15.5± 0.91	10.0 ± 0.85	5.5 ± 0.64^#^
**MEMK (30) + RE (1)**	102.5 ± 5.18^*^	30.5 ± 1.84^*^	11.5 ± 0.64
**MEMK (100) + RE (1)**	82.75± 1.10^*^	22.75± 1.10^*^	6.5 ± 0.28^*^
**MEMK (200) + RE (1)**	75.75 ± 2.01^*^	17.5 ± 1.04^*^	5± 0.40^*^
**Vitamin E (10) + RE (1)**	42 ± 1.08^*^	19 ± 1.29^*^	5.25 ± 0.47^*^
**F**	501.71	80.87	42.72

RE-Reserpine, MEMK-methanolic extract of *M. koenigii* leaves, VCM- vacuous chewing movements, OB-orofacial burst, TP-tongue protrusion. n = 5. The observations are mean ± SEM. ^#^ p < 0.05 compared with vehicle treated group.^*^ p < 0.05 compared with reserpine-treated group. One-way ANOVA followed by Dunnett’s test.

*Drugs, chemicals and solvents*

Reserpine (Sigma, Mumbai) and Haloperidol (Searle, Mumbai) were used for the study. All other chemicals used were of analytical grade.

*Plant material and extraction*

Fresh leaves of *Murraya koenigii *were obtained from local market during the month of September, Nashik and authenticated by Dr. P. G. Diwakar, Botanical Survey of India, Pune. The herbarium was deposited at the Botanical Survey of India, Pune (voucher number MUKKID 1). Leaves of *Murraya koenigii* (1 Kg) were dried, powdered and defatted with the petroleum ether (60-80°C) using Soxhlet’s extractor. Petroleum ether was removed and marc was air dried. Marc was successively extracted with methanol. The methanolic extract was concentrated and evaporated to dryness (% yield: 14% w/w). MEMK was dissolved in water for injection and administered in a volume of 1 mL/Kg orally. Doses and pre-treatment times of the extract were obtained from the preliminary studies in our laboratory.

**Table 2:** Effect of MEMK on locomotor activity in reserpine-treated rats.

**Treatment (mg/Kg, p.o.)**	**No. of squares traversed**	**No. of rearing**
**Vehicle**	35.75 ± 0.85	12.5± 0.64
**RE (1)**	3.75±0.47 ^#^	2.25 ± 0.25 ^#^
**MEMK (200)**	25.75± 0.85^#^	13.5 ± 0.64
**MEMK (30) + RE (1)**	4 ± 0.40	2.25 ± 0.25
**MEMK(100) + RE (1)**	11.5 ± 1.32^*^	6.5± 0.64^*^
**MEMK(200) + RE (1)**	12.5 ± 0.64^*^	7.75 ± 0.62^*^
**Vitamin E (10) + RE (1)**	11.75 ± 0.85^*^	3.5 ± 0.28
**F**	204.21	83.88

RE-Reserpine, MEMK-methanolic extract of *M. koenigii* leaves. n = 5. The observations are mean ± SEM. ^#^ p < 0.05 compared with vehicle-treated group.^*^ p < 0.05 compared with reserpine-treated group. One-way ANOVA followed by Dunnett’s test.

*Phytochemical analysis*

MEMK was subjected to the identification of phytoconstituents using methods as described earlier (4).

*Effect on reserpine-induced orofacial dyskinesia*

Rats were divided in 6 groups, each containing five animals. The rats received, vehicle (0.1% acetic acid solution, SC in a dose of 5 mL/kg), reserpine (1.0 mg/Kg, SC), MEMK (200 mg/Kg, p.o.) *per se*, MEMK (30, 100 and 200 mg/Kg, p.o.) with reserpine (1.0 mg/Kg, SC), and vitamin E (10 mg/Kg, p.o.) with reserpine (1.0 mg/Kg, SC). MEMK and vitamin E were administered 1 h prior to reserpine for 5 days. Reserpine was administered on the 1^st^, 3^rd^ and 5^th^ day.

*Behavioral parameters*

After the injection of reserpine, rats were placed in a Plexiglas observation box (22 cm × 22 cm × 22 cm) for a 10 min habituation period. All rats were observed for 5 min period. An observer blind to the treatment recorded the number of vacuous chewing movements (VCM), tongue protrusion (TP) and orofacial burst (OB) as described by Cousins (5). Then, the effect of MEMK on locomotion was evaluated using the open-field apparatus (6). The total number of squares traversed and number of rearing was counted for 5 min. The effect of MEMK on the catalepsy was determined for 3 h using bar test (6). All these parameters were recorded on the 5^th^ day.

**Table 3:** Effect of MEMK on biochemical parameters in reserpine-treated rats.

**Treatment (Dose in mg/Kg, p.o.) for 5 days**	**SOD (U/mg of wet tissue)**	**Catalase(CAT) (U/mg of wet tissue)**	**LPO (µM/mg of wet tissue)**	**GSH (nmole/mg of wet tissue)**
**Vehicle**	2.35 ± 0.02	2.37 ± 0.01	1.94 ± 0.03	13.82 ± 0.04
**RE (1)**	1.67 ± 0.01^#^	1.69 ± 0.01^#^	16.84 ± 0.38^#^	7.42± 0.02^#^
**MEMK (200)**	2.82 ± 0.04^#^	2.88 ± 0.01	1.54 ± 0.02	18.68 ± 0.05^#^
**MEMK (30) + RE (1)**	1.75 ± 0.01	1.80 ± 0.04*	2.62± 0.03^*^	6.97 ± 0.03^*^
**MEMK(100) + RE (1)**	1.84 ± 0.02^*^	1.85 ± 0.01*	2.51 ± 0.01^*^	11.21 ± 0.07^*^
**MEMK (200) + RE (1)**	2.64 ± 0.02^*^	2.66 ± 0.028*	2.11 ± 0.01^*^	18.37 ± 0.05^*^
**Vitamin E(10) + RE (1)**	1.88 ± 0.03^*^	1.90± 0.07*	5.76 ± 0.08^*^	13.65 ± 0.04^*^
**F**	384.99	396.84	1385.32	10758.04

RE-Reserpine, MEMK-methanolic extract of *M. koenigii* leaves. SOD-superoxide dismutase, LPO-lipid peroxidation, GSH-glutathione reductase. n = 5. The observations are mean ± SEM. ^#^ p < 0.05 compared with vehicle treated group.* p < 0.05 compared with reserpine treated group. One-way ANOVA followed by Dunnett’s test.

*Biochemical parameters*

After the measurement of catalepsy on the 5^th^ day, the animal was sacrificed, the brain was removed, and the forebrain was dissected out and rinsed with isotonic saline and weighed. A 10% (w/v) tissue homogenate was prepared in 0.1 M phosphate buffer (pH = 7.4). The post nuclear fraction for catalase assay was obtained through the centrifugation of the homogenate at 1000 g for 20 min, at 4°C and for other enzyme assays centrifuged at 12,000 *g* for 60 min at 4°C. The supernatant was used for the determination of SOD (7, 8), CAT (9), GSH levels (10), and the extent of LPO (11). Shimadzu-2450 UV spectrophotometer was used for the assessment of SOD, CAT, GSH, and LPO.

*Effect on Haloperidol-induced catalepsy in mice*

Mice in groups of five received vehicle, MEMK (30, 100 and 200 mg/Kg, p.o.), 1 h before the haloperidol (1 mg/Kg, IP). The Duration of catalepsy was measured for 3 h using Bar test (6). The forepaw of mouse was placed on a bar elevated at 3.5 cm and the latency to remove the paw from bar was noted for each animal.

**Table 4:** Effect of MEMK on haloperidol- induced catalepsy in mice.

**Treatment (mg/Kg, p.o.)**	**Duration of catalepsy (sec) after**						
	**0 min**	**30 min**	**60 min**	**90 min**	**120 min**	**150 min**	**180 min**
**HPL(1)**	7.33 ± 0.33	176.7 ± 0.88	184.3 ± 1.20	217.7 ± 1.45	256.7 ± 0.88	214.3 ± 0.33	219.7 ± 0.88
**MEMK (30) + HPL (1)**	5 ± 0.57	105.7 ± 0.66^*^	133 ± 1.52	155 ± 1.73^*^	173 ± 0.57^*^	160.3 ± 0.88^*^	155.3 ± 0.33^*^
**MEMK (100) + HPL (1)**	3 ± 0.57	143 ± 1.52^*^	140 ± 1.45	145 ± 0.88^*^	160 ± 1.15^*^	140 ± 1^*^	133 ± 1.45
**MEMK (200) + HPL (1)**	0.66 ± 0.33	65.33 ± 0.88^*^	77.33 ± 4.96	110 ± 0.57^*^	110 ± 1.15^*^	113 ± 1.52^*^	112.3 ± 0.88^*^
**F**	--	2144.73	252.97	1300.35	3962.95	1754.74	2302.52

HPL - Haloperidol, MEMK- methanolic extract of *M. koenigii* leaves. n = 5. The observations are mean ± SEM. ^*^ p < 0.05 compared with haloperidol-treated group. One-way ANOVA followed by Dunnett’s test.

*Statistical analysis*

The mean ± SEM values were calculated for each group. One-way ANOVA followed by Dunnett’s multiple comparison tests were used for statistical analysis. Value of p < 0.05 was considered statistically significant.

## Results and Discussion

*Phytochemical analysis*

The phytochemical analysis of MEMK revealed the presence of alkaloids, carbohydrate, cardiac glycosides, flavonoids, saponins, tannins and phenolic compounds.

*Effect of MEMK on reserpine-induced orofacial dyskinesia in rats*

The frequency of VCM, TP and OB were significantly increased after an acute treatment with reserpine (1 mg/Kg, SC) compared to the vehicle-treated group. MEMK *per se* does not produce significant change in VCM and OB when compared to the vehicle-treated group. Treatment with MEMK (30, 100 and 200) significantly reversed the reserpine-induced VCM, TP, and OB when compared to the reserpine-treated group (Table 1). Number of squares traversed and rearing was significantly reduced after the reserpine treatment in open-field apparatus when compared to the vehicle-treated group. MEMK *per se* does not produce any significant change in rearing but significantly decreased the number of traversed squares that was observed when compared to the vehicle-treated group. Treatment with MEMK **(**30, 100 and 200) exhibited significant increase in the number of squares traversed and rearing in reserpine-treated group (Table 2).

*Effect of MEMK on SOD levels in the forebrain of reserpine-treated rats*

Reserpine treated rats exhibited decrease levels of SOD in the forebrain homogenates. MEMK *per se* produced significant increase in SOD levels when compared to the vehicle-treated group. The administration of MEMK (30, 100 and 200) significantly reversed the reserpine-induced decrease in forebrain SOD levels in the reserpine-treated rats (Table 3).

*Effect of MEMK on CAT levels in the forebrain of reserpine-treated rats*

Reserpine treated rats showed decreased levels of CAT in the forebrain homogenates. MEMK *per se* produced significant increase in CAT levels when compared to vehicle-treated group. Administration of MEMK (30, 100 and 200) significantly reversed the reserpine-induced decrease in the forebrain SOD levels in reserpine-treated rats (Table 3).

*Effect of MEMK on LPO in the forebrain of reserpine-treated rats*

Reserpine treatment for 5 days induced lipid peroxidation as indicated through significant rise in the forebrain malonaldehyde levels as compared to control rats. MEMK *per se* produced significant decrease in the extent of LPO when compared to the vehicle-treated group. The administration of MEMK (30, 100 and 200) significantly reversed the extent of lipid peroxidation caused by reserpine (Table 3).

*Effect of MEMK on GSH levels in the forebrain of reserpine-treated rats*

The administration of reserpine significantly decreased the forebrain GSH levels. MEMK *per se* produced significant increase in GSH levels when compared to the vehicle-treated group. The administration of MEMK (30, 100 and 200) significantly reversed the haloperidol-induced decrease in the forebrain GSH levels (Table 3).

*Haloperidol-induced catalepsy*

Haloperidol produced catalepsy in mice, which remained for 3 h. When compared with the control group, a significant reduction in catalepsy was observed in the MEMK-treated group (30, 100 and 200) (Table 4).

The brain and nervous system are particularly prone to the free radical damage since the membrane lipids are very rich in polyunsaturated fatty acids and the certain areas of brain are very rich in iron, which favor the generation of free radicals (12). The basal ganglia regions of the brain are highly vulnerable to the free radical overproduction caused via increased dopamine turnover, since they use the elevated amounts of energy and contain considerable amounts of polyunsaturated fatty acids (13).

Chronic treatment with neuroleptics increases free radical production and oxidative stress (14). A role for increased reactive oxygen species and oxidative stress in the etiopathology of neuroleptic-induced TD has been proposed (15-18). Elkashef and Wyatt (19) have reported that rats with vacuous chewing movement had significantly higher thiobarbituric acid reactive substances (TBARS) in the striatum, suggesting the increased lipid peroxidation and free radical production in these animals. The chronic use of neuroleptics is also reported to cause decrease in the activity of antioxidant defense enzymes, superoxide dismutase (SOD) and catalase (20). Previous studies have shown that the use of reserpine (1 mg/Kg) on the alternate day for period of 5 day induced orofacial dyskinesia is a putative model of TD (21).

Reserpine binds tightly to adrenergic vesicles in central and peripheral adrenergic neurons and remains bound for a prolonged period of time. The interaction inhibits the vesicular catecholamine transporter that facilitates the vesicular storage. Thus, nerve ending lose their capacity to concentrate and store norepinephrine and dopamine. Catecholamine leaks into the cytoplasm, where they are metabolized through the interneuronal monoamine oxidase, and lead to the formation of 3,4- dihydroxyphenyacetic acid (DOPAC) and hydrogen peroxide. In presence of ferrous ion, H_2_O_2 _undergoes spontaneous conversion, forming a hydroxyl free radical, causes oxidative stress and degeneration of neuron (22).

Increased frequency of VCM, OB and TP was observed in rats treated with reserpine. Treatment with MEMK (30, 100, 200 mg/Kg, p.o.) significantly inhibited the reserpine-induced VCM, OB and TP. Treatment with MEMK *per se* did not produce any significant change on VCM and OB when compared to vehicle-treated groups.

In this study, a significant decrease in the concentration of SOD and CAT levels was observed in reserpine-treated group. MEMK (100 and 200 mg/Kg) treatment significantly reversed the changes in the antioxidant enzyme levels induced by reserpine treatment. A decrease in the activity of SOD can result in the decreased removal of superoxide ion, which can be harmful to the organs. Moreover, the enhanced SOD activity in MEMK*-*treated group (100 and 200 mg/Kg) might be involved in the scavenging of O_2_ generated from reserpine. There is a general agreement that flavonoids act as the scavengers of reactive oxygen species (23).

Phytochemical screening of MEMK revealed the presence of cardiac glycosides, flavonoids, tannins and phenolic compounds, alkaloids, carbohydrates, and saponins. Flavonoids, tannins and phenolic compounds have antioxidant activity (24). The* in-vivo* antioxidant activity of MEMK may be due to the presence of flavonoids and phenolic compounds.

Haloperidol, a non-selective post-synaptic D_2_-receptor antagonist induces the ataxia in humans (25) and catalepsy in animals (26). Haloperidol-induced catalepsy is also associated with an increase in the oxidative stress in the brain and different stages of catalepsy appear to be directly correlated with the brain histamine content (27). Pretreatment with MEMK dose-dependently inhibited the haloperidol-induced catalepsy which is in the agreement with previous reports (28).

## Conclusions

MEMK dose-dependently protected the reserpine-treated rats against the increase in LPO, decrease in GSH, CAT and SOD levels. MEMK significantly attenuated the reserpine-induced orofacial dyskinesia. It also inhibited the haloperidol-induced catalepsy. Hence, the leaves of *Murraya koenigii* may be useful in the treatment of neuroleptic-induced orofacial dyskinesia.

## Notes

*Declaration of interest*

The authors report no declarations of interest.
